# Special Issue “The Promising Future of CAR-Based Therapies: A Matter of Molecular Details”

**DOI:** 10.3390/ijms26199587

**Published:** 2025-10-01

**Authors:** Fabio Nicolini, Orsola Montini, Matteo Zurlo, Sarah Tettamanti, Massimiliano Mazza

**Affiliations:** 1 Advanced Cellular Therapies and Rare Tumors Unit, IRCCS Istituto Romagnolo per lo Studio dei Tumori (IRST) “Dino Amadori”, 40121 Meldola, Italy; fabio.nicolini@irst.emr.it (F.N.); matteo.zurlo@irst.emr.it (M.Z.); 2 Tettamanti Center, Fondazione IRCCS San Gerardo dei Tintori, 20900 Monza, Italy; orsolamontini91@gmail.com

In the rapidly advancing field of cancer immunotherapy, Chimeric Antigen Receptor (CAR)-T cell therapy is redefining treatment paradigms and offering renewed hope to patients with hematologic malignancies. As the technology progresses, subsequent generations of CAR designs are addressing earlier limitations and broadening therapeutic possibilities to include solid tumors and beyond. This Special Issue provides a comprehensive overview of recent advances in CAR engineering, including logic-gated systems, universal CARs, and genome editing techniques, as well as emerging tools such as AI-guided antibody design and sophisticated biosensors for monitoring cytotoxicity. Collectively, these innovations are paving the way for more precise, effective, and durable immunotherapies, heralding a new era in personalized cancer treatment. In this regard, few innovations have excited scientists and clinicians as much as CAR-T cell therapies. CARs are shaping current and future cancer treatments. The review entitled “Significant Advancements and Evolutions in Chimeric Antigen Receptor Design” by Gaimari and colleagues [1] provides a thorough and timely overview of this innovative area, highlighting the rapid progress in CAR design and extensively discussing its impact on future clinical applications. Originally acclaimed for its success in treating hematological cancers, CAR-T therapy is now gearing up to tackle the major hurdles of solid tumors. This progress is driven by innovative engineering techniques that improve the targeting, durability, and safety of this therapeutic approach. From early-generation CARs to the versatile SUPRA CARs [2,3,4,5], this evolution in design marks a fundamental shift in how we understand and manipulate the immune system. Notably, the review highlights the significance of “logic-gated” CARs—such as Dual Tandem [3,6,7] and SynNotch systems [8,9]—which allow T cells to respond with exceptional accuracy to complex antigenic landscapes. The development of universal CARs (UCARs), which can be activated and controlled through adaptable molecular switches [10], represents a crucial step towards scalable, off-the-shelf therapies. The integration of genome editing tools, such as CRISPR/Cas9 [11,12] and TALENs [12], brings another dimension, allowing precise control over T cell behavior, reducing adverse effects like graft-versus-host disease (GVHD) and improving the feasibility of allogeneic approaches. Innovations in non-molecular mechanisms—such as enzyme-secreting CARs and those responsive to external stimuli like light or small molecules—are equally impressive, pointing towards a future where immunotherapy is not only potent but also intelligent and tunable. Yet, despite this progress, key challenges remain. The tumor microenvironment, antigen heterogeneity, limited CAR T cell persistence and penetration, and manufacturing obstacles remain significant barriers to overcome, especially for solid tumors. In line with this, the review by Wei and colleagues [13] discusses a key challenge in CAR-T therapy: the limited persistence of CAR-T cells, especially in the treatment of solid tumors and relapsed hematological malignancies. Despite notable success in treating B-cell leukemias—achieving complete remission rates of over 80% in some trials [14,15]—many patients eventually relapse due to the poor expansion and survival of infused CAR-T cells [16]. This review emphasizes how CRISPR/Cas9 genome-editing technology provides a potent solution to these issues by reprogramming CAR-T cells to improve their functionality and longevity. By employing targeted gene editing approaches, scientists can create CAR-T cells that possess stem-like memory phenotypes (such as TSCM and TCM), exhibit improved proliferation, and show reduced exhaustion—an impairment mainly caused by persistent antigen stimulation [17]. Notably, disrupting immune checkpoints like PD-1 and LAG3 with CRISPR/Cas9 has demonstrated the ability to rejuvenate exhausted CAR-T cells and prolong their anti-tumor activity [11,18,19,20,21,22,23]. Additionally, transcriptional negative regulators such as Cb1-b [24], PTP1B [25], SOX4, and ID3 [26], and NR4A [27], have been identified as key factors driving exhaustion as their deletion enhances T cell effector functions [27,28]. To further improve their persistence in the human body and over time, CAR-T cells can be modified to resist immunosuppressive cytokines in the tumor microenvironment. For instance, knocking out the TGF-β receptor II prevents TGF-β–induced CAR-T cell exhaustion, leading to increased tumor killing activity in vitro [29] and supporting the development of central and effector memory cell subsets as demonstrated in cell line-derived xenografts or patient tumor-derived xenograft models [30]. Additionally, as the knockdown of CISH and A2AR restores cytokine signaling and enhances CAR-T cell cytotoxicity, as shown in different solid tumor xenograft models [31,32]. Epigenetic reprogramming strategies are also discussed, such as the deletion of TET2, PRDM1, or DNMT3A, which promotes memory phenotypes and reduces exhaustion-related methylation patterns [16,33,34]. CRISPR technology is a promising approach for identifying novel targets that modulate exhaustion and promote memory phenotypes. For example, RASA2 deficiency enhances MAPK signaling, boosting CAR-T cell activation and persistence [35]. Similarly, the disruption of mediator complex components, such as MED12 and CCNC, sustains effector functions and IL-2 responsiveness [36]. Epigenetic regulators, including the cBAF complex subunit Arid1a, have also been implicated in preventing exhaustion and improving tumor control. Effective CAR-T therapy requires robust expansion and sustained cytotoxicity. CRISPR screens have identified key genes that enhance these properties. The overexpression of LTBR, for example, activates the NF-κB pathway, promoting proliferation [37]. Knockouts of TLE4 and IKZF2 reduce exhaustion and boost killing capacity in glioblastoma-specific CAR-T cells [38]. Furthermore, innovative hybrid screening methods have identified membrane proteins such as PDIA3, which, when perturbed, enhance tumor-killing potency [39]. Gain-of-function screens have highlighted metabolic regulators, such as PRODH2, whose overexpression reprograms T cell metabolism to improve long-term anti-tumor activity [40,41]. Allogeneic, or “off-the-shelf,” CAR-T cells derived from healthy donors represent an ideal alternative, offering immediate availability and consistent quality. However, the main challenge for allogeneic CAR-T cells clinical success is GVHD, caused by the presence of donor T cell receptors (TCRs) able to recognize recipient antigens, and graft rejection triggered by recipient immune responses against donor human leukocyte antigen (HLA) molecules. CRISPR/Cas9 gene editing offers a powerful approach to overcome these barriers by precisely disrupting TCR and HLA class I expression in donor T cells. Preclinical and clinical studies have demonstrated that such modifications significantly reduce the risk of GVHD and graft rejection, enabling the development of universal CAR-T cells that can be safely administered to any patient without the need for personalized manufacturing [42,43,44]. Besides enhancing immune compatibility, CRISPR/Cas9 technology also allows for the precise and strategic improvement of CAR-T cell functionality by modulating cytokine signaling. Cytokines such as IL-15 and IL-18 have been shown to bolster CAR-T cell persistence and anti-tumor activity. IL-23, in particular, promotes granzyme B production, reduces inhibitory PD-1 expression, and enhances CAR-T cell expansion [45,46,47]. The clinical translation of these insights is already progressing. Most current clinical trials involving CRISPR/Cas9-edited CAR-T cells are in early phases (Phase I), primarily focusing on universal CAR-T cells engineered to avoid immune rejection and GVHD. A notable study by Ottaviano et al. [48] utilized CRISPR/Cas9 to delete the TRAC and CD52 genes in CD19-targeted CAR-T cells administered to pediatric patients with relapsed or refractory B-cell acute lymphoblastic leukemia (B-ALL). Despite some manageable adverse events—including grade II cytokine release syndrome and grade IV neurotoxicity with skin GVHD—these complications were effectively managed, highlighting the safety of this approach. Similarly, a trial targeting the dual antigens CD19 and CD22 in B-ALL patients disrupted the TRAC and CD52 genes to develop universal CAR-T cells. Remarkably, 83.3% of patients reached complete remission by day 28 post-infusion, demonstrating strong clinical evidence for the effectiveness of CRISPR-engineered CAR-T cells in blood cancers [38,48]. In summary, this review emphasizes the transformative potential of CRISPR/Cas9 based systems in advancing CAR-T cell therapy. By optimizing differentiation, reversing exhaustion, and enhancing resistance to immunosuppressive signals, genome editing significantly boosts the persistence and anti-tumor activity of CAR-T cells. Although off-target effects and immunogenicity remain technical challenges, ongoing improvements in delivery methods and sgRNA design point to promising future developments. CRISPR/Cas9 technology is evolving rapidly and is poised to become a crucial tool for developing next-generation, durable cancer immunotherapies. The development of next-generation nucleases (such as dCas9, Cas9 nickases, Cas12, Cas13 and many others), along with innovative genome editing platforms like base editing and prime editing, has markedly enhanced both precision and safety by substantially reducing off-target effects. These technological breakthroughs are critically contributing to the refinement of genome editing tools, thereby facilitating a more efficient and reliable transition toward clinical application. Another important aspect is the evaluation and monitoring of CAR-T cells’ cytotoxicity; innovative methods have been identified to provide convenient readouts of target cell destruction by effector cells. Bednar and colleagues [49] developed a genetically encoded fluorogenic biosensor for visualizing granzyme B activity (GZMB), which is useful for observing T cell cytotoxicity. A new fluorescent biosensor, called the cleavage-responsive sensor for T cell activity level (CRSTAL), has been developed to this end. CRSTAL, activated by GZMB and caspase-8, was tested in stable cell lines, demonstrating a strong and persistent fluorescent signal. Upon antigen recognition by T cells, the exocytosis of cytotoxic granules, such as GZMB, mediates target cell destruction. The ability to detect GZMB activity, therefore, has significant implications for monitoring CAR-T cell activity. The CRSTAL sensor is designed with self-splicing inteins and a GZMB recognition sequence to specifically trigger fluorescence upon GZMB cleavage. This advanced design aims to minimize background fluorescence and ensure a clear signal. The authors examined CRSTAL activation and functionality using transfected cells, which demonstrated an increase in fluorescence when exposed to active GZMB (GZMBΔGE). CRSTAL activation was confirmed in doxycycline-induced cell lines, and its effectiveness was tested in monitoring CAR-T cell activity. CRSTAL-293T-CD19 target cells exhibited an approximately 2.2-fold increase in fluorescence upon co-incubation with CAR-T cells, indicating an apoptosis signal and confirming that CRSTAL can be used in vitro as an indicator of cell-mediated cytotoxicity to assess the efficacy of cell therapies. The CRSTAL biosensor overcomes the limitations of conventional cytotoxicity assessment methods. Traditional approaches rely on flow cytometric analysis of T cell activation marker expression, measurement of secreted cytokines linked to T cell activation, or the assessment of cell death via the release of Lactate Dehydrogenase (LDH), luciferase, 51Cr, or europium. These methods have significant disadvantages, including being indirect and nonspecific, as well as involving radioactive isotopes. Potential applications in animal models are proposed, using either genetically modified mice expressing CRSTAL or xenograft models implanted with reporter-expressing target cells. Regarding clinical monitoring of GZMB activity, it is only feasible through ex vivo experiments. Alternative strategies have been described, such as using a quencher as a fluorescent sensor, but this still needs to be clinically tested [50]. In summary, the method for detecting T cell cytotoxicity via the CRSTAL biosensor represents a significant advancement in directly monitoring GZMB-mediated cytotoxicity. Its high specificity, sensitivity, and stability make it particularly useful for CAR-T cell research and broader immunological studies providing a reliable measure of Cytotoxic T Lymphocytes (CTL) or Natural Killer (NK) cell effector function. 

While tools like CRSTAL are enhancing the precision with which CAR-T cell activity and efficacy can be measured, complementary efforts are urgently needed to improve the monitoring of CAR-T cell-associated toxicities, which can be severe and multifaceted. 

CAR-T cell therapy has transformed the treatment landscape for B-cell hematologic malignancies, offering promising outcomes for diseases such as diffuse large B-cell lymphoma, follicular lymphoma, and multiple myeloma. While its efficacy is well established, increasing attention has been directed towards the spectrum of associated toxicities. Among them, cytokine release syndrome (CRS) and immune effector cell-associated neurotoxicity syndrome (ICANS) are widely recognized and closely monitored in clinical settings. However, thrombotic events (TEs) remain underreported and poorly characterized [51]. In this multicenter retrospective study, Schorr and colleagues [52] analyzed 140 patients treated with FDA-approved CAR-T cell therapies to investigate the incidence and characteristics of TEs in this population. The study found that 7.14% of patients developed clinically significant thromboses, including pulmonary embolisms, deep vein thromboses, a cerebral venous sinus thrombosis, and one case of arterial thrombosis manifesting as non-ST elevation myocardial infarction. These events occurred a median of 23.5 days post-infusion, with considerable variation in timing. The most robust correlates of thrombosis were elevated D-dimer levels—90% of affected patients exhibited peaks of D-dimer levels three times higher than the upper standard limit—and the presence of ICANS, which was significantly associated with TE development (*p* = 0.04). In contrast, no significant associations were found with CRS severity, coagulation parameters such as PT, aPTT, or fibrinogen, or with established risk prediction scores, including Padua and ISTH DIC. These findings suggest a mechanistic link between neurotoxicity and endothelial activation in the pathogenesis of post-CAR-T thrombosis. The authors propose that incorporating D-dimer surveillance and ICANS grading into clinical practice may improve early identification and prophylactic management of thrombosis risk. As the therapeutic use of CAR-T cells expands, this study underscores the need to refine toxicity monitoring and develop evidence-based strategies to mitigate adverse effects, offering new insights into an evolving safety profile that warrants further prospective validation. While clinical studies such as that by Schorr et al. highlight the urgent need to monitor and mitigate CAR-T-related toxicities, a parallel line of research is focused on improving the very foundation of CAR design to enhance efficacy and reduce risk at the molecular level. In this regard, the recent study by Martarelli and colleagues [53] represents a significant advance in cancer immunotherapy, focusing on the rational design of CARs targeting CD30, a key marker in certain lymphomas [54,55]. Their innovative method integrates artificial intelligence (AI)-driven molecular docking with steered molecular dynamics (SMD) simulations to precisely select single-chain variable fragments (scFvs) that exhibit optimal binding features, streamlining the selection process prior to CAR-T cell engineering [56,57]. This combination of computational techniques addresses a key challenge in CAR development, where traditional experimental screening of scFv antibodies is time-consuming, expensive, and labor-intensive. Using AI-guided molecular docking, the authors virtually screened multiple anti-CD30 monoclonal antibody clones, predicting their binding affinities and interaction modes with the target antigen. The inclusion of SMD simulations offered dynamic insights into the stability and unbinding forces of the scFv–antigen complexes, capturing molecular flexibility and solvent effects that are not captured by static docking predictions. This combined in silico strategy enabled a more refined evaluation of candidate scFvs, reducing the dependency on extensive laboratory testing [58,59]. Importantly, the computational predictions demonstrated a strong correlation with experimental validation, including surface plasmon resonance (SPR) assays and functional CAR-T cell tests performed both in vitro and in vivo [60,61]. This agreement confirms the robustness and dependability of the AI-SMD pipeline as a substitute for early-stage scFv screening. The method not only speeds up the CAR-T development process but also has the potential to lower costs and minimize the use of animal models, thereby aiding in the faster translation of these therapies into clinical practice [62]. Martarelli et al.’s work illustrates the increasing influence of AI in immunotherapy design, reflecting a broader trend where machine learning and physics-based modelling work together to improve antibody engineering and drug discovery. The combination of AI-driven docking with molecular dynamics simulations creates new opportunities for developing more accurate and personalized CAR constructs, with the aim of enhancing therapeutic efficacy and safety. Future efforts may involve expanding this computational framework to target other tumor antigens and integrating advanced AI tools such as AlphaFold-based structural predictions and deep learning-enhanced docking algorithms to further boost accuracy. Pairing these approaches with high-throughput experimental validation will be crucial for establishing standardized and efficient pipelines for CAR design. In summary, the study by Martarelli and colleagues represents a significant advancement in the application of AI and molecular simulations for the development of CAR-T cell therapy. Their approach establishes a valuable precedent for future efforts in computational immunoengineering, with the potential to speed up the discovery and optimization of new immunotherapeutic agents for precision oncology [63]. Hanssens and colleagues [64] highlight the complexity of predicting the success of idiotype-specific VHH-based CAR-T cell therapy for multiple myeloma (MM), emphasizing the importance of selecting VHHs specifically for the intended application.

Notably, although two CAR-T products targeting B-Cell maturation Antigen (BCMA) have already been approved for the treatment of multiple myeloma [65], relapse remains a significant concern in a subset of patients [66].

Relapse can be caused by immune responses against CAR-T cells, particularly if the scFv has a non-human origin and turns out to be immunogenic. This can lead to premature T cell exhaustion and the upregulation of immunosuppressive markers, receptor clustering and tonic signaling. To restrain the limitations related to immune responses, the authors used variable heavy domains of heavy chain (VHHs), derived from camelid heavy-chain-only antibodies, instead of scFvs. VHHs are relatively small and resemble human VH-III family domains, which makes them less immunogenic than murine-derived scFvs. Regarding the limitations related to BCMA, several alternative MM targets are under evaluation [67], and Hanssens et al. have developed anti-B-cell receptor (BCR) idiotype VHHs, although their patient specificity limits applicability. 

The authors compared the efficacy of different VHH CAR constructs in side-by-side experiments and realized that, despite diverse VHHs showing similar and strong affinity for the antigen and targeting MM cells in vitro, significant differences in IL-2 secretion were observed when expressed on CAR T cells. In summary, the study demonstrated the importance of in vitro functional tests for the attentive, case-by-case selection of an antigen-binding moiety, as its functionality can change when expressed in a CAR on T cells. This work also suggests that strategies such as ML or AI, as previously described by Martelli et al., may be highly effective in narrowing down VHH candidates given a known target antigen.

To conclude, CAR-based therapies are undergoing a profound transformation, driven by the synergistic integration of molecular engineering, genome editing, biosensing technologies, and artificial intelligence. This Special Issue highlights not only the extraordinary progress made in refining CAR constructs—from logic-gated systems and universal CARs to CRISPR-enhanced persistence and AI-guided design—but also underscores the multidisciplinary efforts that are accelerating their clinical translation.

While significant challenges remain—particularly in targeting solid tumors, overcoming immunosuppressive microenvironments, and ensuring long-term efficacy—the wealth of preclinical and clinical evidence presented suggests that these barriers can be addressed in the future. The application of next-generation tools, such as genome editing for functional reprogramming, biosensors for real-time cytotoxicity monitoring, and in silico design platforms, is shifting the paradigm towards more intelligent, tunable, and personalized immunotherapies. Importantly, this evolving field calls for continued collaboration among molecular biologists, bioengineers, clinicians, and data scientists to refine these technologies and ensure their safety, scalability, and accessibility. As we stand at the threshold of a new era in precision oncology, the detailed mechanistic insights and technological innovations described herein lay the groundwork for a future in which CAR-based treatments can be rapidly tailored, safely deployed, and broadly effective against a wide range of cancers.
